# Ankle-Brachial Index as the Best Predictor of First Acute Coronary Syndrome in Patients with Treated Systemic Hypertension

**DOI:** 10.1155/2020/6471098

**Published:** 2020-07-17

**Authors:** Wojciech Myslinski, Agata Stanek, Marcin Feldo, Jerzy Mosiewicz

**Affiliations:** ^1^Department of Internal Medicine, Medical University of Lublin, Staszica 16, 20-081 Lublin, Poland; ^2^Department of Internal Medicine, Angiology and Physical Medicine, Faculty of Medical Sciences in Zabrze, Medical University of Silesia, Batorego 15 St., 41-902 Bytom, Poland; ^3^Department of Vascular Surgery and Angiology, Medical University of Lublin, Staszica 16, 20-081 Lublin, Poland

## Abstract

**Objective:**

The objective of our study was to evaluate the incidence of target organ damages (TOD) in patients with arterial hypertension and the first ever episode of myocardial infarction (N-STEMI or STEMI) and to determine which of the analyzed kinds of TOD had the highest predictive value for the assessment of the likelihood of acute coronary syndrome (ACS). *Material and Methods*. The study group consisted of 51 patients with treated systemic hypertension, suffering from the first episode of myocardial infarction (N-STEMI or STEMI), confirmed by coronary angiography and elevation of troponin. The control group consisted of 30 subjects with treated hypertension and no history of myocardial ischaemia. In all subjects' measurements of blood lipids, hsCRP and eGFR were measured. TOD, such as intima-media thickness (IMT), presence of atherosclerotic plaques, ankle-brachial index (ABI), and left ventricular hypertrophy, were assessed.

**Results:**

Age, BMI, blood pressure, and time since diagnosis of hypertension did not differ between the study groups. There were no differences regarding blood lipids and eGFR, while hsCRP was significantly increased in the study group. The left ventricular mass index was similar in both groups. Patients with myocardial infarction had significantly increased IMT and decreased ABI. The statistical analysis revealed that only ABI was the most significant predictor of ACS in the study group.

**Conclusion:**

Among several TOD, ABI seems to be the most valuable parameter in the prediction of ACS.

## 1. Introduction

Arterial hypertension is one of the most important modifiable risk factors for cardiovascular complications [1]. In clinical practice, we often find that the extent of cardiovascular damage implies that the actual duration of arterial hypertension is longer than that declared by the patient, possibly due to the long-term asymptomatic course of the disease as well as the cooccurrence of other risk factors for cardiovascular diseases. Therefore, in addition to the presence of risk factors, an important role in global cardiovascular risk assessment is played by identification of subclinical target organ damage (TOD) [2]. TOD such as left ventricular hypertrophy (LVH), atherosclerotic plaque, carotid intima-media thickness (IMT), ankle-brachial index (ABI), pulse wave velocity (PWV), and renal injury features are the best determinants of the condition of the cardiovascular system [3]. In the ESC/ESH guidelines, each TOD has equivalent weight, meaning that slight thickening of the intima-media complex has the same score as advanced left ventricular hypertrophy or hemodynamically significant stenosis within the carotid or lower limb arteries [1].

The objective of our study was to evaluate the incidence of TOD in patients with arterial hypertension and the first ever episode of myocardial infarction (N-STEMI or STEMI) and to determine which of the analyzed kinds of TOD had the highest predictive value for the assessment of the likelihood of acute coronary syndrome.

## 2. Material and Methods

### 2.1. Study Subjects

The study was conducted in a group of 51 patients aged 47 to 80 years, including 32 men and 19 women with arterial hypertension who had experienced their first acute coronary episode. Study group inclusion criteria included the age of ≤80 years, typical anginal pain or changes in ECG records, increased troponin I or T levels, and no history of previous acute coronary syndrome (ACS) episodes. Coronary angiography was performed in all patients confirming the presence of coronary lesions; the time elapsed since the ACS was not longer than 4 days. All patients had been diagnosed with arterial hypertension. The diagnosis of arterial hypertension was based on the use of 1 or more antihypertensive drugs. The average age in the study group was 64.8 years, including 69.6 years in women and 61.9 years in men. The mean BMI was 27.7 kg/m^2^; overweight or obesity was diagnosed in 34 (66.6%) patients, including 16 (31.37%) patients diagnosed with obesity. Thirteen (25.49%) patients smoked, and 14 (27.45%) received antidiabetic treatment.

The control group consisted of 30 subjects aged 45 to 78, including 16 men and 14 women treated for arterial hypertension with no history of episodes of myocardial infarction. Just as in the study group, the diagnosis of arterial hypertension was based on the use of 1 or more antihypertensive drugs. The average age in the study group was 64.2 years, including 68.5 years in women and 60.4 years in men. The mean BMI was 28.4 kg/m^2^; overweight or obesity was diagnosed in 22 (73.3%) patients, including 12 (40.0%) patients with BMI of 30 kg/m^2^ or higher. There were 9 (30%) smokers in the control group. Antidiabetic treatment was received by 11 (36.6%) subjects.

As it was not possible to unambiguously determine the duration of arterial hypertension, time since the diagnosis of arterial hypertension was taken into account and was similar in both groups.

All subjects in the study and the control group received statins as part of primary or secondary prevention.

Qualified patients were informed of the study objectives and expressed their consent to participate. The study was approved by the Bioethics Committee at the Medical University of Lublin.

The characteristics of the study groups are given in Table 1.

### 2.2. Biochemical Analysis

#### 2.2.1. Laboratory Parameters

Blood samples of all the subjects were collected in the morning before the first meal. Samples of whole blood (5 ml) were collected from the basilic vein into tubes containing ethylenediaminetetraacetic acid tripotassium salt (Sarstedt, S-Monovette with 1.6 mg/ml EDTA-K_3_) and into tubes with a clot activator (Sarstedt, S-Monovette).

Total cholesterol, HDL cholesterol, and LDL cholesterol (T-Chol, HDL-Chol, and LDL-Chol) and triglyceride (TG) concentrations in serum were estimated using routine techniques (COBAS INTEGRA 400 plus analyzer, Roche Diagnostics, Mannheim, Germany). Concentrations were expressed in mg/dl. The inter- and intra-assay coefficients of variations (CV) were, respectively, 2.8% and 5.4% for T-Chol, 3.2% and 5.4% for HDL-Chol, 2.6% and 6.5% for LDL-Chol, and 2.5% and 7.6% for TG. Estimated GFR (eGFR) and hsCRP levels were also determined in all patients.

### 2.3. Estimation of the Ankle-Brachial Index

A Vivid 4 ultrasound system equipped with a 7–10 MHz adjustable frequency vascular transducer was used for ABI assessments. The ABI values were measured after a 5-minute rest in a recumbent position. At the first stage, a color Doppler technique was used to visualize blood flow within the dorsal artery of the foot or, in cases of visualization problems, within the posterior tibial artery. Next, the cuff of a mercury sphygmomanometer previously placed above the ankle of the right lower limb was inflated until the blood flow in the visualized artery stopped. Upon slow deflation of the sphygmomanometer cuff, the value of systolic blood pressure at which the return of blood flow was observed in the examined artery was recorded. In order to verify the measured systolic pressure value, a second measurement was made using the pulsed-wave Doppler method to assess the return of blood flow. Immediately after the color Doppler and pulsed-wave Doppler measurements were completed within the lower limb, the value of systolic blood pressure at which blood flow returned to the right brachial artery was also recorded. Similar measurements were made on the arteries of the left lower limb and the left upper limb. ABIs were calculated as ratios of the systolic pressure values for ipsilateral lower and upper limb arteries. Separate ABI values were determined for color Doppler and pulsed-wave Doppler measurements. Lower ABI values were used for statistical purposes, with subjects presenting with ABI > 1.3 not being included in statistical analysis [4].

### 2.4. Echocardiography

Echo scans were acquired on a Vivid 4 ultrasound system with a 2 MHz transducer. During the scan, patients were lying on their left sides. The transducer was placed above the 4th intercostal space near the left edge of the sternum to produce a 2D image of the heart in the parasternal longitudinal view. The following cardiac chamber size and wall thickness measurements were made in M-mode to evaluate the left ventricular mass:

LVEDD: left ventricular end diastolic dimension (mm),

IVSD: interventricular septal thickness at end diastole (mm),

PWD: posterior left ventricular wall thickness at end diastole (mm).

The left ventricular mass (LVM) was calculated using the following formula[5, 6]:

(1) $$\begin{array}{c} \text{LVM}=1.04\times \left[\left(\text{LVEDD}+\text{IVSD}+\text{PWD}\right)3-\text{LVEDD}3\right]-13.6 \end{array}$$

The left ventricular mass index (LVMI) was calculated by dividing the left ventricular mass (in grams) by the body surface area (in square meters).

### 2.5. Estimation of Intima-Media Thickness

The final stage of the study consisted of ultrasound measurements of the carotid intima-media thickness (IMT). Measurements were made using a Vivid 4 ultrasound system with a 7–10 MHz vascular transducer. During the examination, patients were lying on their backs with their heads facing backwards and away from the examination side.

IMT was assessed at the common carotid artery, carotid bulb, and internal carotid artery on the right and the left. Due to better repeatability, measurements were made on distal arterial walls. First, a measurement was made within the common carotid artery about 2 cm distally from the bifurcation site; then, measurements were made at bifurcation and within the internal carotid artery 2 cm proximally from the carotid bulb. The average IMT was calculated separately for the left and the right side from all the above measurements. The presence of atherosclerotic plaque was also assessed, with 1.5 mm being taken as the minimum atherosclerotic plaque thickness.

### 2.6. Statistical Analysis

Statistical analysis was carried out using the SPSS 14 software package for the MS Windows operating system.

The chi-square compliance test was performed to verify any statistically significant differences in the numbers of subjects within the study groups. The same test was used to verify whether the compared groups differed in terms of the incidence of comorbidities, smoking, and atherosclerotic plaque being present in the carotid arteries.

The Mann-Whitney *U* test (unequal sample sizes) was performed to assess whether subjects from the study group differed from those in the control group in terms of measurement variables.

At the next stage of the statistical analysis, potential significant correlations between the study variables were verified using Pearson's correlation coefficient.

The final step consisted in a logistic regression analysis with myocardial infarction status as the dependent variable and the measurement variables as predictors (quantitative scale).

Differences at the significance level of *p* < 0.05 were considered statistically significant.

## 3. Results

The Mann-Whitney *U* test was performed in order to verify whether the patients with the history of ACS differed from those with no history of coronary incidents in terms of measurement variables.

Patients with the history of ACS were found to present with increased hsCRP levels, decreased ABI values, and carotid intima-media complex thickness. The results of the statistical analysis of the results are presented in Table 2.

**Figure 1:**
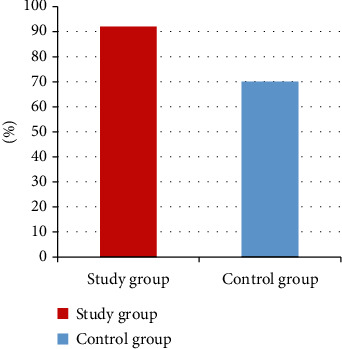
Prevalence of atherosclerotic plaque in carotid arteries (*p* = 0.021).

A statistically significant difference in the incidence of atherosclerotic plaque was observed. In the study group, the presence of one or more atherosclerotic plaque(s) was observed in 47 patients (92.15%) as compared to 21 patients (70%) in the control group (Figure 1).

The mean LVMI value in the study group was 120.49 ± 32.21 g/m^2^ as compared to 113.53 ± 25.19 g/m^2^ in the control group; the difference was not statistically significant. Significant gender-specific LVMI differences were also sought as different normal LVMI values had been adopted for men and women. No statistically significant differences were observed for the examined variables between the study groups. Results of echocardiographic LVH assessments are presented in Table 3.

Pearson's correlation analysis was used to check whether there were any statistically significant relationships between the examined variables.

**Figure 2:**
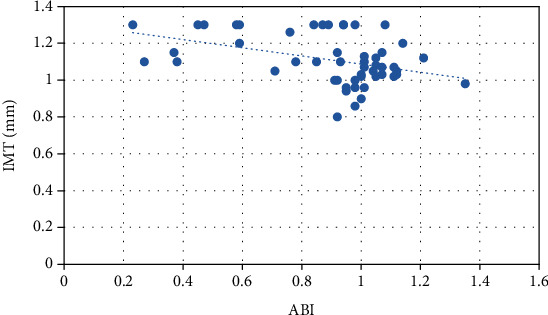
Pearson's correlation between IMT and ABI (*r* = −0.40, *p* < 0.05).

The analysis of Pearson's correlation coefficients for the study group revealed a negative correlation between ABI and IMT (*r* = −0.40, *p* < 0.05). Data are presented in Figure 2.

In order to determine potential correlations between the study variables and the occurrence of myocardial infarction, the Logistic Regression Variable Selection Method was used with myocardial infarction status as the dependent variable and the measurement variables as predictors. Due to the small number of subjects, predictors were entered into the model using the forward selection (Wald) method.

They proved to match the data well: *χ*^2^ = 9.77 and *p* = 0.282. It explained approximately 27.8% of the observed variance of the dependent variable. The model introduced two predictors, CRP and ABI, in two steps (Table 4).

The statistical analysis revealed that only ABI was an important predictor of ACS in the study group. The Exp(*B*) factor indicates that higher ABI values reduce the likelihood of ACS.

## 4. Discussion

Along with strokes, acute coronary syndromes belong to the most dramatic episodes in the long-term process of atherosclerosis. The risk factors and organ-related complications associated with higher cardiovascular risk are well defined in the current guidelines of European and American cardiac and hypertension societies [1]. Cardiovascular risk assessment scales included in these guidelines are based on population-based studies and facilitate the classification of patients into different risk categories. Organ-related complications of arterial hypertension included in the global cardiovascular risk assessment, i.e., left ventricular hypertrophy, carotid intima-media thickness, ankle-brachial index, and renal injury features, are taken into account as equivalent to one another. This means that a patient with extensive atherosclerotic lesions within the carotid arteries and reduced ABI is categorized into the same group as a patient with mild left ventricular hypertrophy and eGFR of 50 ml/min/1.73 m^2^. The intuitive evaluation of both patients, however, suggests that particular attention should be paid to the patient presenting with features of the extensive atherosclerotic process in the imaging studies. Therefore, it is interesting to answer the question of which kinds of target organ damage due to arterial hypertension can be best used to differentiate the group of patients who have experienced an ACS episode from the group of patients who have hitherto not experienced such an episode.

The main objective of the study was to answer the question of whether any differences can be found in the intensity of asymptomatic target organ damage between the two study groups otherwise homogeneous in terms of anthropometric variables and the incidence of risk factors and comorbidities. Another objective of the study was to identify a potential target organ damage parameter characterized by the highest ACS predictive strength. The selected parameters of target organ damage, highly valued in clinical practice mainly due to their ease of use as well as to the availability of diagnostic tools such as echocardiography and vascular ultrasound, were assessed.

As confirmed by the results, the analysis of generally accepted cardiovascular risk factors such as diabetes, smoking, lipid disorders, overweight, or obesity may by itself not be sufficient for a reliable evaluation of ACS risk level. Therefore, the current risk assessment scales have been expanded to include modules that take into account the presence of target organ damage and comorbidities [7].

As shown by the analysis of the study results, the study and the control groups differed in a statistically significant manner in terms of ABI, carotid IMT, and IMT/ABI ratio values. A statistically significant difference in the incidence of atherosclerotic plaque within the carotid arteries was identified; the difference, however, did not apply to the average thickness of atherosclerotic plaque. Another finding which emerged following the analysis of the measurement variables was that subjects with a history of myocardial infarction presented with significantly higher levels of C-reactive protein compared to subjects within the control group, suggesting the involvement of inflammation within the vascular wall and atherosclerotic plaque. However, no statistically significant intergroup differences were observed concerning the LVMI values.

In order to determine the factor(s) most strongly associated with ACS, a logistic regression analysis was performed with myocardial infarction status as the dependent variable and measurement variables as predictors. As shown by the statistical method used for that purpose, ABI values of <0.9 are associated with high risk of ACS. The second predictor, albeit of a low prognostic value, was the elevation of C-reactive protein levels.

Numerous studies showed that reduced ABI is associated with the presence of atherosclerotic lesions in the carotid and coronary arteries. According to Criqui and Denenberg, as well as to Dormandy et al., the incidence of coronary artery disease in patients with ABI < 0.9 ranges from 20 to 60% if the diagnosis was based on physical examination, history, and electrocardiogram to as much as 90% in patients subjected to angiographic diagnostics [8–10]. Unfortunately, the sensitivity of ABI is too low for the presence of, e.g., coronary disease to be excluded on the basis of its correct value alone. On the other hand, ABI is specific enough to indicate elevated cardiovascular risk at low ABI values. Therefore, the presence of atherosclerotic lesions within the lower extremity arterial bed expressed as ABI of <0.9 should not be considered an isolated disease but rather an indicator of similar lesions being present in other parts of the circulatory system [8]. The relationships between peripheral arterial disease and cardiovascular mortality are independent of age, BMI, smoking, LDL and HDL cholesterol levels, blood pressure, glycemia, or the history of symptoms of angina pectoris, myocardial infarction, and stroke [11, 12].

However, no explanation is provided by the above data as to why the low ABI values rather than, for example, carotid intima-media thickening had the highest correlation with the incidence of ACS in our study group. In addition, the mean ABI in the study group was 0.89 and thus was only slightly below the value decisive for confirmation or rejection of PAD diagnosis. Perhaps the answer to this question can be provided by demands made by some researchers regarding the arbitrary character of the ABI threshold for the diagnosis of peripheral arterial disease. In normal conditions, systolic blood pressure at the ankle level is 8–15% higher than that at the shoulder level, and therefore, ABI values are considered to be normal when greater than 1.0. In the MESA, SHS, and CHS studies where ABI values in ranges 0.9–0.99 and 1.0–1.09 were considered, respectively, as borderline and low normal, these values were also shown to be associated with an increased risk of death for cardiac reasons [12–14]. The results of these studies suggest that despite the absence of the diagnosis of peripheral arterial disease, patients with ABI values between 0.9 and 1.09 are nonetheless burdened with mild to moderate atherosclerosis which indicates a risk of lesions being present, e.g., in the coronary arteries. In the light of the aforementioned results from large studies, it appears that the stage of atherosclerosis within the lower limb arteries, and therefore in the coronary arteries, of the study group patients was much higher than that suggested by the ABI value used to define peripheral arterial disease (PAD).

An attempt to demonstrate the relationship between ABI and the extent of lesions in the coronary arteries was made by Papamichael et al. [15]. They performed coronary angiography examinations and calculated ABI values in 165 patients to assess the advancement of lesions in patients with stable coronary disease. The results of this study provided evidence for the existence of correlation between reduced ABI and the extent of lesions in coronary arteries. Of 44 people with three-vessel disease as diagnosed by angiography, 13 had ABI of <0.9; for the sake of comparison, only 4 out of 37 subjects with confirmed one-vessel disease presented with ABI values providing the grounds for the diagnosis of peripheral arterial disease. The logistic regression analysis carried out by the study authors led to the final conclusion that ABI of <0.9 was a predictor of cardiovascular incidents. This result coincides with our findings obtained in a much smaller group. However, does identification of a correlation between low ABI and three-vessel disease justify coronary angiography being performed on a routine basis in each patient with ABI < 0.9? While the answer remains unclear, it seems that such patients should be subject to special monitoring, active screening for other kinds of TOD, and possible qualification for noninvasive studies, such as angio-CT of the coronary arteries.

Another factor identified in the logistic regression analysis as being correlated with the risk of ACS was the elevated concentration of C-reactive protein. For many years, the CRP levels have been associated with the presence of generalized inflammation. CRP is synthesized mainly in the liver, but also in smooth muscles, including those within the walls of the coronary arteries [16]. Ridker et al. claim that of all the acute phase proteins, elevated CRP levels have the strongest association with elevated cardiovascular risk [17]. Pasceri and other researchers demonstrated that C-reactive protein directly contributes to the initiation of the atherogenesis by inducing adhesion molecules on the endothelial cell, opsonizing LDL molecules for their subsequent absorption by macrophages leading to the formation of foam cells, and stimulating and activating monocytes to produce various growth tissue factors [18]. Goldstein et al. observed that elevated CRP levels as determined during hospitalization due to ACS were related to a larger extent of coronary atherosclerotic lesions, increased risk of death, and higher incidence of recurrent myocardial infarction and need for revascularization [19]. These observations were consistent with those obtained in a multicenter study conducted in 1773 patients with acute coronary syndrome. Patients with CRP > 10 mg/l had significantly higher risk of death within 30 days of the coronary incident, regardless of troponin levels [20]. There are 3 levels of risk for cardiovascular events based on hsCRP concentration, namely, low risk for hsCRP < 1.0 mg/l, medium risk for hsCRP of 1.0 to 3.0 mg/l, and high risk for hsCRP > 3 mg/l. On the basis of data from the FHS study, Wilson et al. concluded that only hsCRP > 3.0 mg/l is associated with increased cardiovascular risk [21]. However, it should be noted that the mean CRP levels in our control group were higher than 3 mg/l. Perhaps higher cut-off values should be adopted for prognostic purposes as observed in the study by Oltrona et al. [20].

When evaluating the results of our study, we also decided to verify whether the combined use of two hypertension-related target organ damage markers, i.e., ABI and carotid IMT, would differentiate the study group from the control group in a statistically significant manner. The analysis using the Mann-Whitney *U* test revealed an intergroup difference at the significance level of *p* = 0.001 which means that the IMT/ABI ratio was significantly lower in the control group as compared to the study group. At present, it is difficult to conclude whether the predictive value of this “complex” parameter could be higher than the predictive value of either parameter assessed separately. No mention has been found in the available literature on the use of complex risk assessment markers that would simultaneously describe the stage of atherosclerosis at different levels of the arterial bed. Instead, attempts were made to create a scoring system based on ABI and IMT values. On the basis of their research, Hayashi et al. assumed that correct ABI and IMT values would be assigned the score of 0 while abnormal values would be assigned the score of 1. Next, they divided their study population into 3 groups depending on the score. A total score of 0 was assigned when the patient had presented with unremarkable ABI and IMT values, a total score of 2 was assigned when both markers were abnormal, and a total score of 1 was assigned when only one of the marker values was unremarkable. The conclusion stemming from the use of this simple scoring system was that the incidence of cardiovascular diseases was statistically significantly higher in subjects who scored 1 or 2 points [22]. This means that the lesion on the arterial bed should be sought at many levels, as identification of their absence in only one part of the cardiovascular system may lead to incorrect evaluation of a patient's condition and, therefore, to a failure to implement appropriate management.

The results are indicative of the high value of ABI measurements in the assessment of ACS risk in patients with arterial hypertension. Notably, none of the subjects within the study or the control group reported any signs of intermittent claudication or had previously undergone diagnostic screening for peripheral arterial disease. This is due to the fact that the symptomatic course of the disease is observed in patients with very advanced atherosclerotic lesions due to the development of collateral circulation. Postexercise drops in systolic blood pressure values as observed in exercise tests are even better for identification of disturbed supply of blood to the lower limbs [23]. Perhaps postexercise ABI values would be the strongest predictor of ACS in our study group. However, sensitive postexercise assessment of blood pressure within the lower limb arteries is also a method which cannot be used in general clinical practice, particularly in primary care.

With no doubt, very interesting information could be provided by prospective observation of patients with arterial hypertension and by identification of target organ damage parameter that would, either alone or combined, ensure the best prediction of future coronary episodes. It cannot be ruled out that acute coronary episodes would occur in the near future in all control group subjects. However, it is important to demonstrate that despite the same risk factors, arterial beds may differently respond to the same damaging stimuli, obviously as a consequence of individual traits. Thus, “individualization” of cardiovascular risk assessments becomes particularly important today. Further studies are needed to assess the predictive strength of individual TOD parameters, possibly facilitating the development of new scales which would differentiate the predictive values of individual TOD parameters. Our study shows that from among numerous easily measurable TOD parameters, the ankle-brachial index has a very high ACS predictive value. Therefore, as well as for the ease of its estimation, it should be particularly recommended for practitioners who encounter the problem of identifying high cardiovascular risk patients in their everyday practice.

## 5. Conclusions

Our study originated from doubts regarding the equivalence of target organ damage parameters as used in the assessment of cardiovascular risk. From among numerous kinds of total organ damage examined in the study, the ankle-brachial index was the only strong predictor of acute coronary syndrome in our study population.

## Figures and Tables

**Table 1:** General characteristics of the study group and the control group subjects.

	Study group (*n* = 51)	Control group (*n* = 30)	*p*
Mean age (years)	64.84 ± 9.83	64.2 ± 9.34	NS
Mean age, females (years)	69.6 ± 8.73	68.5 ± 6.02	NS
Mean age, males (years)	61.9 ± 9.33	60.4 ± 10.23	NS
BMI (kg/m^2^)	27.73 ± 5.06	28.47 ± 5.04	NS
Diabetes	14 (27.45%)	11 (36.6%)	NS
Time since the diagnosis of hypertension (years)	8.54 ± 6.82	8.37 ± 6.9	NS
Smokers	13 (25.49%)	9 (30%)	NS
Systolic blood pressure (mmHg)	129.3 ± 8.2	127.1 ± 7.8	NS
Diastolic blood pressure (mmHg)	77.7 ± 6.1	79.2 ± 6.4	NS

NS: nonsignificant.

**Table 2:** Results of measured laboratory and ultrasonography variables.

Parameters	Study group (*n* = 51)	Control group (*n* = 30)	Mean range (study group)	Mean range (control group)	*p*
hsCRP (mg/l)	14.70 ± 23.81	5.78 ± 9.26	47.19	30.48	0.002
Total cholesterol (mg/dl)	186.39 ± 40.65	174.23 ± 42.75	40.65	42.75	0.231
LDL cholesterol (mg/dl)	111.57 ± 29.5	116.13 ± 61.6	41.74	39.75	0.714
Triglycerides (mg/dl)	141.96 ± 80.96	131.23 ± 111.14	43.82	36.20	0.159
T-Chol/HDL cholesterol	4.20 ± 1.15	3.75 ± 1.41	44.75	34.62	0.061
eGFR (ml/min)	83.22 ± 31.91	78.74 ± 30.39	42.12	39.10	0.577
ABI	0.89 ± 0.25	1.05 ± 0.13	34.68	51.75	0.002
IMT (mm)	1.11 ± 0.13	1.00 ± 0.18	46.31	31.97	0.008
IMT/ABI	1.45 ± 0.87	0.96 ± 0.2	47.44	30.05	0.001
Mean plaque thickness (mm)	2.27 ± 0.99	1.79 ± 1.34	43.33	37.03	0.243

The average thickness of plaque or plaques (if more than one is present) was taken into account for calculation purposes.

**Table 3:** Mean LVMI values in the study and control groups according to gender.

	LVMI (study group) (g/m^2^)	LVMI (control group) (g/m^2^)	*p*
Females	112.63 ± 26.15	106.79 ± 22.32	0.689
Males	125.16 ± 34.87	119.44 ± 26.75	0.562

**Table 4:** Predictors of acute coronary syndrome.

ACS predictors (study group)	*B*	Wald	*p*	Exp(*B*)
hsCRP	0.05	3.14	0.076	1.055
ABI	-5.89	7.15	0.007	0.003

## Data Availability

All data are included in the tables within the article.
